# Survey of anesthesiologists’ practices related to steep Trendelenburg positioning in the USA

**DOI:** 10.1186/s12871-018-0578-5

**Published:** 2018-08-21

**Authors:** Fouad G. Souki, Yiliam F. Rodriguez-Blanco, Sravankumar Reddy Polu, Scott Eber, Keith A. Candiotti

**Affiliations:** Department of Anesthesiology, Jackson Memorial Hospital, University of Miami/Jackson Health System, 1611 NW 12th Ave, DTC 318, Miami, FL 33136 USA

**Keywords:** Laparoscopy complications, Steep Trendelenburg, Positioning injuries, Trendelenburg complications, Anesthesia practices, Anesthesia survey

## Abstract

**Background:**

Steep Trendelenburg during surgery has been associated with many position-related injuries. The American Society of Anesthesiology practice advisory recommends documentation, frequent position checks, avoiding shoulder braces, and limiting abduction of upper extremities to avoid brachial plexopathy. We conducted a web-based survey to assess anesthesiologists’ practices, institutional policies, and complications encountered when using steep Trendelenburg.

**Methods:**

Two thousand fifty randomly selected active members of the American Society of Anesthesiology were invited via email to participate in a 9-item web-based survey. Results are reported as absolute numbers and proportions with 95% confidence interval (CI).

**Results:**

Survey response rate was 290 of 2050 (14.1%). 44.6% (95% CI, 38.9–50.3) of the respondents documented anesthesia start and finish, 73.9% (95% CI, 68.8–79) frequently checked positioning during surgery, 30.8% (95% CI, 25.4–36.2) reported using shoulder braces, 66.9% (95% CI, 61.5–72.3) tucked patients’ arms to the side, 54.0% (95% CI, 48.2–59.8) limited fluid administration, and more than two-thirds did not limit the duration or inclination angle. Notably, 63/290 (21.7%) reported a complication and only 6/289 (2.1%) had an institutional policy. The most common complication was airway and face edema, second was brachial plexus injury, and third was corneal abrasions. Most institutional policies, when present, focused on limiting duration of steep Trendelenburg and communication with surgical team. Only 1/6 policies required avoiding use of shoulder braces.

**Conclusion:**

Based on survey results, practices related to steep Trendelenburg varied among USA anesthesiologists. Differences included protective measures, documentation, positioning techniques, fluid management, and institutional guidelines. The singular commonality found among all respondents was lack of institutional policies. Survey results highlighted the need for institutional policies and more education.

## Background

First linked by name to the 19th century German surgeon Freidrich Trendelenburg, steep Trendelenburg positioning describes the head-down tilting (25°-45°) of an otherwise supine patient [1, 2]. This mode of positioning provides optimal field exposure for a wide variety of surgeries performed using laparoscopic, robotic, or other techniques [2, 3]. However, it is associated with physiological changes and complications that span the majority of organ systems [3–5].

Part of the anesthesiologist’s responsibilities is to document, examine, adjust, and ensure adequate patient positioning during surgery. Careful intraoperative positioning may reduce frequency and severity of position-related adverse events [2]. Although the American Society of Anesthesiology (ASA) and others have issued recommendations about patient positioning during steep Trendelenburg, there is limited published information as to what constitutes routine practice [6–8].

In the wake of an upper extremity neurologic complication due to robotic surgery at our institution, we conducted a web-survey to obtain data regarding anesthesiologists’ practices when using the steep Trendelenburg position, complications encountered, and institutional policies implemented.

## Methods

We obtained institutional research board approval from the Human Subject Research Office at University of Miami Miller School of Medicine. Participation in the survey implied consent. Using SurveyMonkey^R^ (Portland, OR), we developed a web-based survey following guidelines to survey research in anesthesiology [9]. The survey was pilot tested with 20 members of the anesthesiology department. Sample calculation was based on the assumption that a 95% confidence level with 5% confidence interval (CI) required around 400 responses [10, 11]. Assuming a 20% response rate, 2000 survey invitations were needed.

After reviewing our proposal, the ASA member services sent an email invitation with a brief explanation and survey link to 2050 randomly selected active members. Following 6 weeks of data collection, a second email reminder was sent to the same group. SurveyMonkey^R^ prevented duplication of responses by allowing only one response per electronic device behind the Internet Protocol address. To maintain confidentiality and anonymity, the investigators had no access to the email addresses and the answers could not be traced back to the participants. Non-delivered email was not reported to the investigators, and non-responders could not be identified.

The survey consisted of four close-ended questions, and five open-ended questions (Appendix). The closed-response questions inquired about the limits enforced when using the steep Trendelenburg position for surgery (steepness of head-down angling, duration of positioning), occurrence of complications, and availability of institutional policies. The latter two questions employed logic to proceed to the next related question based on a “yes or no” response. In the open-response questions, respondents were asked to “choose all that apply” and/or provide a free-text answer with regards to measures taken to decrease the duration of the Trendelenburg position, protective equipment and techniques used for positioning, ways to decrease complications, nature of complications encountered, and parameters of institutional policies. None of the survey questions inquired about respondents’ demographic information.

Statistical analysis was performed using the normal approximation method, Wald’s method, to calculate the 95% CI for the proportions [12]. Survey results are reported as absolute values and proportions. The 95% CI was computed for proportions of interest.

## Results

### Survey response rates and speed

We received 290 responses out of 2050 email invitations, a 14.1% response rate. We obtained 54.8% (159/290) of the total responses after the first email invitation. Nearly all survey responses arrived within two weeks of each email invitation (> 96%). Survey completion rate was 99.6%; only one respondent did not complete the survey through to the last question.

### Anesthesiologist practices

When asked about inclination angle during steep Trendelenburg position, 32.8% (95% CI, 27.4–38.2) (95/290) of respondents picked “I do not limit the inclination angle” and 40% (95% CI, 34.4-45.6) (116/290) chose “minimum angle for optimal surgical access”. On the other hand, 69.2% (95% CI, 63.9–74.5) (200/289) did not limit the duration of steep Trendelenburg position.

As for measures taken to minimize duration of steep Trendelenburg position, 68.5% (95% CI, 63.2–73.8) (198/289) reported having a discussion with the surgeon, 44.6% (95% CI, 38.9–50.3) (129/289) documented the start and finish, 15.9% (95% CI, 11.7–20.1) (46/289) stated that they provided the surgeon with an hourly reminder, and 14.2% (95% CI,10.2–18.2) (41/289) took no action.

The most common technique used to position patients during steep Trendelenburg and prevent sliding off operating table was the use of a gel mattress (61.9% [177/286]). Other techniques employed were waist straps (36.0% [103/286]), gel or foam pads across the shoulders (33.2% [95/286]), shoulder braces (30.7% [88/286]), shoulder to hip strapping (29.7% [85/286]), bent knees (15.4% [44/286]), wrist straps (12.2% [35/286]), and ankle cuffs (2.4% [7/286]). Open-ended replies included bean bag (5.6% (16/286)), egg crate foam mattress (4.2% (12/286)), and slightly elevating patient’s back (0.7% (2/286)).

To avoid complications related to positioning, 73.9% (95% CI, 68.8–79.0) (212/287) repeatedly assessed the patient’s position during surgery, 66.9% (95% CI, 61.5–72.3) (192/287) tucked the patient’s arms to the sides, 54.0% (95% CI, 48.2–59.8) (155/287) avoided excess fluid administration, and 44.6% (95% CI, 38.8–50.4) (128/287) avoided abduction, external rotation or extension of upper extremities. Moreover, 30.7% (95% CI, 25.3–36.1) (88/287) avoided the use of shoulder braces and wristlets, 28.9% (95% CI, 23.7–34.1) (83/287) of respondents limited the angle, and 19.2% (95% CI, 14.6–23.8) (55/287) limited the duration of steep Trendelenburg position. Other comments included monitoring ventilation, application of saline ointment to the eyes, and monitoring of renal perfusion pressure.

### Complications

Sixty-three respondents out of 290 (21.7% (95% CI, 17.0–26.4)) reported encountering one or more complication related to Trendelenburg positioning. In total, 91 complications were reported (Table 1). The most common complication was airway and face edema (39.5% [36/91]), second was brachial plexus injury 16.4% (15/91), and third was corneal abrasions (13.1% [12/91]).

**Table 1:** Complications of Trendelenburg position reported by 63 out of 290 participants

Complications of Trendelenburg position	Number (%)
Airway and/or facial edema	36 (40%)
Brachial plexus injury	15 (17%)
Corneal abrasion	12 (13%)
Patient sliding off table	8 (9%)
Lower extremity nerve injury	6 (7%)
Visual loss or defect	3 (3%)
Compartment syndrome	3 (3%)
Respiratory problems	3 (3%)
Shoulder symptoms (pain, rotator cuff)	2 (2%)
Alopecia	1 (1%)
Subcutaneous emphysema	1 (1%)
Wrist edema	1 (1%)
Total	91 (100%)

Some participants reported more than one complication. Values are number (proportion)

### Institutional policies

Only 2.1% (6/289) reported having a policy for Trendelenburg positioning. Policies included: minimizing duration of head-down positioning (5/6), frequent discussion with surgeons regarding patient’s positioning (5/6), minimizing inclination angle (3/6), frequent assessments and documentation of patient’s position (3/6), avoiding excessive intravenous fluid administration (2/6), and avoiding shoulder braces (1/6).

## Discussion

While practice habits vary, it is important for anesthesiologists to be aware of their peers’ routines along with existing evidence related to steep Trendelenburg positioning. Significant circulatory and respiratory perturbations have been associated with head-down tilt position alone or in combination with pneumoperitoneum [13–15]. Mean arterial blood pressure (MAP), mean pulmonary arterial pressure (MPAP), as well as right and left ventricular filling pressures (i.e., central venous pressure [CVP], and pulmonary capillary wedge pressure [PCWP]) increase markedly during head-down tilt [13–15]. While cardiac output (CO) is unaffected [13–15] or decreased [16], left ventricular diastolic function is impaired [17]. Respiratory wise, peak and mean inspiratory pressures are increased by pneumoperitoneum and exacerbated after Trendelenburg positioning. On the other hand, venous admixture does not change and lung compliance decrease by almost 50% of the initial value [15, 18].

Surgery-related risk factors during steep Trendelenburg leading to position injuries include operative time and how the patient is situated on the operating table [6, 19]. Published reports reveal that prolonged steep Trendelenburg positioning increases risk of postoperative morbidity in patients undergoing robotic surgery for gynecologic malignancy and urologic procedures [19–21]. Lithotomy positioning for more than 2 h has been associated with an increased risk of nerve injuries [22]. During radical prostatectomies, rhabdomyolysis has been shown to occur with excessive lithotomy position and prolonged operative times (> 5 h) [23, 24]. Facial edema has also been associated with prolonged operative times [20] due to the larger amounts of fluids administered [25]. In this regards, half of survey respondents avoided excess fluid administration. Governed mainly by surgical needs and surgeons experience, more than two-thirds of anesthesiologists reported not limiting the duration or inclination angle of steep Trendelenburg.

The ASA practice advisory recommends limiting abduction of the arms to less than 90° and avoiding shoulder braces in the steep Trendelenburg position to decrease risk for brachial plexus neuropathy [7]. In a case series, Devarajan emphasized that the arms should be adducted and tucked to the patient’s side with the avoidance of shoulder girdle restraints to decrease brachial plexopathy during steep Trendelenburg positioning [8]. An ASA survey conducted in 2000 showed that two-thirds agreed with the opinion that the use of shoulder braces influences the risk of peripheral neuropathy [7]. In our survey, more than two-thirds of respondents tucked patients’ arms to the side; however, one-third still reported using shoulder braces.

The ASA task force consensus is that periodic perioperative assessments ensure maintenance of the desired position and documentation helps the practitioner focus attention on relevant aspects of patient positioning [7]. In this survey, three-fourths of respondents frequently checked patients’ positioning and only half documented the start and finish of Trendelenburg positioning.

Existence of institutional policies related to Trendelenburg positioning might help in guiding healthcare practitioners and ultimately decrease adverse events. The policies reported in this survey, though scarce, reflected variation and lack of agreement with some of the ASA recommendations; avoiding shoulder braces and arm abduction. However, respondents’ policies emphasized communication with surgical team and decreasing duration of steep Trendelenburg.

The survey highlighted the different complications that may be encountered during steep Trendelenburg positioning and their relative frequency (Table 1). While there is no data published about the overall rate of complications due to steep Trendelenburg positioning, there are published reports about isolated complications (neuromuscular, ophthalmic) [3, 5, 25, 26]. In this survey, the rate of neuromuscular injuries 7.93% (23/290) and corneal abrasions 4.13% (12/290) were comparable to published rates of neuromuscular injuries (5–6.6%) and corneal abrasions (3%) due to steep Trendelenburg positioning [3, 5, 25–28]. This may help validate survey results. Nevertheless, one cannot neglect the possibility of selection and recall bias. Anesthesiologists who had experienced a complication due to steep Trendelenburg positioning may have been more inclined to participate in this survey and report a complication. On the other hand, some anesthesiologists may have under-reported a complication simply because they did not remember or know about it postoperatively.

The survey had some limitations. First, the participants’ demographics related to years of practice, experience (academic or private practice), anesthetic subspecialty, and types of surgeries performed in steep Trendelenburg were not obtained. This data could have helped create associations between some of the responses and the demographics of respondents. Nevertheless, it does not undermine the responses obtained. Second, the response rate (14.1%) was low although the survey was short, anonymous, and a reminder to participate was sent. A low response rate underscores the possibility of non-response bias that could have selected those with strong attitudes towards the subject or who have encountered complications due to steep Trendelenburg. Non-response could also be due to nondelivered email, unfamiliarity with topic, lack of motivation to participate, or neglect of surveys in general. The anonymous nature of the survey prevented us from contacting non-responders to address this potential issue.

Whether there was a difference between responders and non-responders cannot be determined. However, when a survey has less than a 100% response rate, assumptions that the data are “missing at random” holds that responders and nonresponders are not qualitatively different with respect to the outcome measures of interest in the survey [29]. Some reports have called into question the assumed correspondence between nonresponse rate and response bias [29, 30]. Groves et al. found a poor overall correlation between the nonresponse rate and differences between respondents and non-respondents [31].

Despite the nonresponse rate, the number of respondents was enough to have narrow CI results. Since 290 anesthesiologists responded, the maximum half width 95% CI for the proportions was narrow: +/− 5.75%. This was only 0.75% CI difference from our study aim of 5% CI. 

## Conclusion

Based on survey results, practices related to steep Trendelenburg varied among USA anesthesiologists. Differences included protective measures, documentation, positioning techniques, fluid management, institutional guidelines, and position-related complications. Survey results highlighted lack of institutional policies and need for more awareness.

## Appendix

### Survey Questionnaire

1. What is your angle limit when a patient is in steep Trendelenburg?
  - 30 degrees or less
  - 40 degrees or less
  - 45 degrees or less
  - Minimum angle for surgical access
  - I do not limit the angle
2. What is your time limit for a patient in steep Trendelenburg (25–45 degrees of Trendelenburg)?
  - 1 h
  - Less than 2 h
  - Less than 3 h
  - Less than 4 h
  - Less than 5 h
  - I do not have a limit on the duration of steep Trendelenburg
3. What precautions do you personally take to minimize the duration of steep Trendelenburg? Choose all that apply.
  - I remind the surgeon every hour
  - I keep a note in my record when Trendelenburg started and finished
  - I discuss it with the surgeon to remove the patient from Trendelenburg as soon as possible
  - I do not take any precautions
4. What technique do you use to prevent patients from sliding off the table during steep Trendelenburg positioning? Choose all that apply.
  - Gel mattress
  - Shoulder braces
  - Patient strapping: cross-torso method of shoulders to opposite hips
  - Gel or foam pads across the shoulders
  - Waist strap
  - Wrist straps
  - Ankle cuffs
  - Bent knees with ankle restraints
  - None
  - Other (please specify)
5. What precautions do you take to avoid complications related to steep Trendelenburg position?
  - Avoid abduction, external rotation, or extension of the upper extremities
  - Tuck the arms to the patient’s sides
  - Avoid use of shoulder braces and wristlets
  - Repeatedly assess the patient’s position during surgery
  - Limit the angle of Trendelenburg
  - Limit the duration of surgery
  - Avoid excess fluid administration
6. Have you had any complication related to Trendelenburg positioning?
  - Yes
  - No
7. If there was any complication related to Trendelenburg positioning, what was it? Choose all that apply.
  - Brachial plexus injury
  - Lower extremity nerve injury
  - Corneal abrasion
  - Visual loss or defect
  - Airway edema
  - Patient sliding off the operating table
  - Never had any complications related to Trendelenburg
  - Other(please specify):
8. Does your institution have a written policy regarding steep Trendelenburg positioning?
  - Yes
  - No
9. If your institution has a written policy regarding steep Trendelenburg, choose all that apply.
  - Avoid shoulder braces
  - Secure upper extremities to the side of the patient (adduction)
  - Avoid excess IV fluids
  - Minimize the duration of steep Trendelenburg
  - Minimize the angle of steep Trendelenburg
  - Frequent assessment and documentation of adequate position and pressure points
  - Interval discussion with surgeon concerning need for head-down positioning

## Abbreviations

ASA: American Society of Anesthesiology
CI: Confidence interval
CO: Cardiac output
CVP: Central venous pressure
MAP: Mean arterial blood pressure
MPAP: Mean pulmonary arterial pressure
PCWP: Pulmonary capillary wedge pressure
